# Prognostic value of Maspin protein level in patients with triple negative breast cancer

**DOI:** 10.1038/s41598-024-53870-y

**Published:** 2024-07-10

**Authors:** Renan Gomes do Nascimento, Mércia Patrícia Ferreira da Conceição, Daniel Rodrigues de Bastos, Cynthia Aparecida Bueno de Toledo Osorio, Rossana Verónica Mendoza López, Eduardo Moraes Reis, Otto Luiz Dutra Cerqueira

**Affiliations:** 1 Center for Translational Research in Oncology, Cancer Institute of the State of São Paulo (ICESP), Clinical Hospital Faculty of Medicine, University of São Paulo (HCFMUSP), São Paulo, SP 01246-000 Brazil; 2 Department of Clinical Pharmacy and Oncology, Hospital São Camilo (HSC), São Paulo, SP 02401-300 Brazil; 3 Department of Pathological Anatomy, A.C. Camargo Cancer Center, São Paulo, SP 01509-900 Brazil; 4 Departmento de Bioquímica, Instituto de Química, Universidade de São Paulo, São Paulo, SP 05508-900 Brazil

**Keywords:** Biomarkers, Triple negative, Breast cancer, Tissue microarray, Maspin, Biochemistry, Biomarkers, Molecular medicine

## Abstract

The search for prognostic markers in breast cancer has bumped into a typical feature of these tumors, intra and intertumoral heterogeneity. Changes in the expression profile, localization of these proteins or shedding to the surrounding stroma can be useful in the search for new markers. In this context, classification by molecular subtypes can bring perspectives for both diagnosis and screening for appropriate treatments. However, the Triple Negative (TN) subtype, which is already the one with the worst prognosis, lacks appropriate and consistent molecular markers. In this work, we analyzed 346 human breast cancer samples in tissue microarrays (TMA) from cases diagnosed with invasive breast carcinoma to assess the expression and localization pattern of Maspin and their correlation with clinical parameters. To complement our findings, we also used TCGA data to analyze the mRNA levels of these respective genes. Our data suggests that the TN subtype demonstrates a higher level of cytoplasmic Maspin compared to the other subtypes. Maspin transcript levels follow the same trend. However, TN patients with lower Maspin expression tend to have worse overall survival and free-survival metastasis rates. Finally, we used Maspin expression data to verify possible relationships with the clinicopathological information of our cohort. Our univariate analyses indicate that Maspin is related to the expression of estrogen receptor (ER) and progesterone receptor (PR). Furthermore, Maspin expression levels also showed correlation with Scarff-Bloom-Richardson (SBR) parameter, and stromal Maspin showed a relationship with lymph node involvement. Our data is not consistently robust enough to categorize Maspin as a prognostic marker. However, it does indicate a change in the expression profile within the TN subtype.

## Introduction

Breast cancer is the most diagnosed malignancy and the one with the highest mortality rates in women worldwide^1,2^. In Brazil, not unlike global data, it is the most incident tumor with an estimate of approximately 66,280 new cases in 2022^3,4^. Breast cancer is a complex, heterogeneous and multifactorial disease, and can be classified by the profile of gene expression or immunophenotyping in different intrinsic subtypes: Luminal A (ER+ PR+ HER2− Ki-67 < 14%), Luminal B (ER+ PR+/− HER2 ± Ki-67 ≥ 14%), HER2+ (ER− PR− HER2+ Ki-67 > 20%) and Triple Negative (TN) (ER− PR− HER2− Ki-67 > 30%)^5,6^.

Despite great advances in the molecular classification of breast cancer, a major challenge in clinical oncology has been the complete understanding of the mechanisms of intertumoral and mainly intratumoral heterogeneity^7,8^. In addition, multiple drug resistance (MDR) has been considered the biggest obstacle in the systemic treatment of breast cancer, making the disease often uncontrollable and leading to high mortality rates^9^. Consequently, hundreds of other candidates for biomarkers have been investigated for potential implications for diagnosis, prognosis, and prediction of therapeutic response^10^.

The SERPIN superfamily comprises serine protease inhibitors and is divided into 16 different classes^11^. The gene *SERPINB5* encodes the protein Maspin, Mammary serine protease inhibitor, that can act in different cellular processes, such cell adhesion, migration, epithelial mesenchymal transition (EMT) and modulation of gene transcription, depending on different ligands and subcellular locations^12–14^. Described as a tumor suppressor due to experimental evidence of a relationship between its expression and inhibition of tumor growth, invasion, metastasis, and a better prognosis in different cancer types^15–18^. On one hand, in the cell membrane it can inhibit invasion and motility of tumor cells^19,20^, yet translocation to nucleus can also regulate gene transcription and favor tumor suppression^21–24^.

In the present study, we evaluated Maspin expression immunohistochemically, associated its expression levels with clinicopathological features and prognosis of resected breast cancer patients and performed in silico analyses using breast cancer patient’s public data.

## Material and methods

### Patients and tumor specimens

This study was based on a cohort of 346 breast tumor samples, fixed in formalin and embedded in paraffin, obtained from the archive of the Department of Pathological Anatomy of the A.C. Camargo Cancer Center, São Paulo, Brazil, between 1980 and 2005. Inclusion criteria were as follows: Confirmatory histological diagnosis of invasive ductal or lobular breast carcinoma, female gender, availability of demographic data, clinical and pathological information, details of treatment and clinical follow-up. Supplementary Table 1 shows the main clinical characteristics of the studied cohort. Cases that exhibited only lesions in situ, tissue nuclei with < 10% of representative tumor cells, worn or nonexistent spots, patients who received neoadjuvant chemotherapy combined or not with radiotherapy, in addition to samples from male patients were excluded. This study was evaluated and approved by the Research Ethics Committee (CIPE—Centro Internacional de Pesquisa e Ensino—AC Camargo Cancer Center) with protocol number 1822/13. All patients agreed with an informed consent document.

### Construction of tissue microarrays (TMAs)

Briefly, the specimens of breast tumor tissues embedded in paraffin were recovered, sectioned, and stained with H&E (hematoxylin–eosin) to select viable and morphologically representative areas, as described previously^25^. 1 mm cylindrical samples were extracted from the donor blocks using a manual tissue matrix (Tissue microarrayer—Beecher Instruments, Silver Spring, MD, USA) and inserted in order in the recipient blocks. Subsequently, 4 μm sections were cut with a microtome from each TMA block (Tissue Microarray) on adhesion slides for subsequent IHC staining.

### Immunohistochemistry (IHC) staining

Immunohistochemistry (IHC) for detection of candidate proteins for biomarkers in breast cancer was performed as previously described^25–28^. Maspin IHC staining were performed after confection of TMAs. Briefly, the slides were deparaffinized and rehydrated with a decreasing concentration of ethanol, and antigenic recovery was performed using sodium citrate buffer pH 6.0 in 95 °C steam, as previously described by Norton^29^. Then, the slides were treated with proteinase K and blocked with 3% hydrogen peroxide in phosphate buffered saline (PBS) at pH 7.4 for 5 min and washed 3 times with PBS, before adding the primary antibodies of interest. To standardize IHC staining, the dilution of antibodies and the visualization system were optimized in tissue sections archives. TMA’s slides were stained with the following primary antibody anti-Maspin, (BD, #554,292, 1:250 dilution). After, mouse secondary antibody HRP (Horseradish peroxidase) coupled dextran polymer detection system (Advance TM HRP link—Dako) was incubated for 30 min at room temperature after standard washes accordingly to manufacturer recommendations^25^. The presence of Maspin was finally detected by addition of 3,3-diaminobenzine tetrachloride (FLEX DAB + Substrate Chromogen System, Dako) to the samples, as previously described^26–29^. All slides were contrasted with Harris Hematoxylin. All immunostained slides were digitized on the Aperio ScanScope CS platform (Aperio Technologies, Inc., Vista, CA, USA) with 20 × magnification.

### Immunomarking evaluation

All image analyses were performed using the digital pathology software QuPath (Quantitative Pathology & Bioimage Analysis, v0.2.0-m1, University of Edinburgh, United Kingdom)^30^. The quantitative analysis of nuclear and cytoplasmic expression for Maspin were evaluated and scored on tumor cells, in addition, thinking that this protein can be secreted into the extracellular environment, we carry out additional analyses evaluating the tumor microenvironment, in this way, we evaluate and score this marker in the surrounding stroma^31^. All analyses of the immunohistochemical expression of Maspin were performed without prior knowledge of the clinical details of our cohort and the clinical outcome of patients, according to REMARK guidelines^32^. In summary, the flow of our work comprised the estimation of scores for each marker, TMA design, cell segmentation, computation of characteristics and identification of cell type with intensity classification, and all stages were assisted by an experienced pathologist. All TMAs were identified using the “dearrayer” tool with the attribution of the grids and the map with the ID of each imported case. All tissue nuclei were detected using the “simple tissue detection” tool so that all tissues considered insufficient (< 10% of representative tumor cells in the core), with dominant artifacts or consisting only of ductal carcinoma in situ, were removed from our analysis^25^. In addition, for cases that were considered evaluable, but which presented confusing objects and folds of tissue, they were removed manually at this stage. The cells were identified using the custom “cell detection” algorithm^31,33^. A cell classifier was trained separately for each marker evaluated in the study, defining the tumor cell class, stroma, necrosis, and inflammatory infiltrate^31^. The expression rates of each marker were calculated as the percentage of the number of positive cells in relation to the total number of tumor cells^31,34^. The detection classifier for our marker was applied to all datasets. At the end, the samples were divided into two groups (low and high) based on the median values obtained from the H-score for cytoplasmic and nuclear labeling in tumor cells and stromal labeling of Maspin IHC. The following H-score medians were obtained for the Maspin: Cytoplasm 71.07, Nucleus 145.84 and Stroma 19.32, and subsequently, Kaplan Meyer (KM) curves were elaborated according to follow up patients’ data.

### In silico analyses

For compare expression of *SERPINB5* gene, that express Maspin protein, in cancer and normal samples we use the online tool UALCAN (http://ualcan.path.uab.edu)^35^. The Firehose Legacy TCGA public data was accessed through cBioPortal platform (https://www.cbioportal.org/)^36,37^ and mRNA Maspin levels of 1108 patients were downloaded. Excluded from the study were cases of male patients (16 cases) and cases that did not have expression information (4 cases), resulting in a total of 1088 evaluable patients. After performing the exclusion criteria, the expression data were cross-checked with the clinical-pathological data accessed through the UCSC Xena platform (http://xena.ucsc.edu/)^38^ to analyze the association of *SERPINB5* gene expression with different clinical and pathological parameters. The integration of data from both databases was achieved by leveraging the patients' ID/barcode, a shared identifier across the spreadsheets. Median gene expressions were computed, serving as the basis to classify groups as Low (≤ median) or High (> median). Subsequently, the contingency table was formulated, employing the chi-squared test or Fisher's exact test as appropriate. The survival curve adopted the same "high" and "low" stratification, utilizing the Kaplan–Meier (KM) method and log-rank test. For a comprehensive quantitative analysis of *SERPINB5* expression profiles, an initial assessment of Gaussian distribution was conducted. Following this, the variables underwent either the t-test or Mann–Whitney test for paired variables, or ANOVA or Kruskal–Wallis for three or more groups. The beta value (HM450) refers to the DNA methylation level of individual CpG sites in comparison to the corresponding mRNA expression. The presentation of Maspin levels involved log-transformed mRNA expression z-scores, compared to the expression distribution of all samples (RNA Seq V2 RSEM) as previously described^36,37^.

Survival curves were also constructed using downloaded TCGA data. The median expression of the studied genes was used as a cut-off for classification into low or high expression.

### Cell cultures and western blot

The human breast cancer cell lines MCF-7, (luminal) and MDA-MB-231 (Triple Negative) and HS578T were grown in Dulbecco’s Modified Eagle Medium, (DMEM Invitrogen) with 10% fetal bovine serum (Invitrogen) and the non-malignant MCF-10A cells were grown in DMEM/F-12 (Invitrogen) with 10 µg/mL insulin, 100 ng/mL cholera toxin, 500 ng/mL hydrocortisone, 20 ng/mL Epidermal growth factor (Sigma), and 5% horse serum (Invitrogen). All cells were kept in a humidified 5% CO2 incubator at 37 °C and cultured until 70% confluence before subculture or protein extraction. Protein extraction, SDS-PAGE and Western blot were previously described^25^.

### Statistical analysis

Categorical data were assessed using Pearson's chi-square test or Fisher's exact test. Fisher was applied when 25% or more of the expected value was less than 5. Statistical analyzes of the box plots were performed using the t-student or Mann–Whitney, depending on whether the data had a normal distribution or not, respectively. Cumulative survival probabilities were estimated using the Kaplan–Meier method and survival curves were compared using the log-rank test. The survival times for overall survival (OS) were calculated from the date of surgery until the moment of death or last follow-up and for disease-free survival (DFS), the outcome was at the date of cancer recurrence after surgery or end of the follow-up. A statistically significant difference was predetermined for values of p < 0.05. IBM SPSS Statistics (version 23.0; SPSS Inc., Chicago, IL, USA) was used for our analyses.

### Ethics approval and consent

This study was evaluated and approved by the Research Ethics Committee (CIPE—Centro Internacional de Pesquisa e Ensino—AC Camargo Cancer Center) with protocol number 1822/13 and registered at the research regulatory platform of the Brazilian Ministry of Health (CAAE: 20373713.8.0000.5432). Clinical samples used in this study were retrieved from the A.C. Camargo Cancer Center biorepository and all participants signed an approved informed consent form.

### Good practices statement

We attest that all methods performed were performed in accordance with institutional guidelines and regulations, as well as good laboratory practices.

## Results

### Expression profile of maspin in human breast tumors

**Figure 1:**
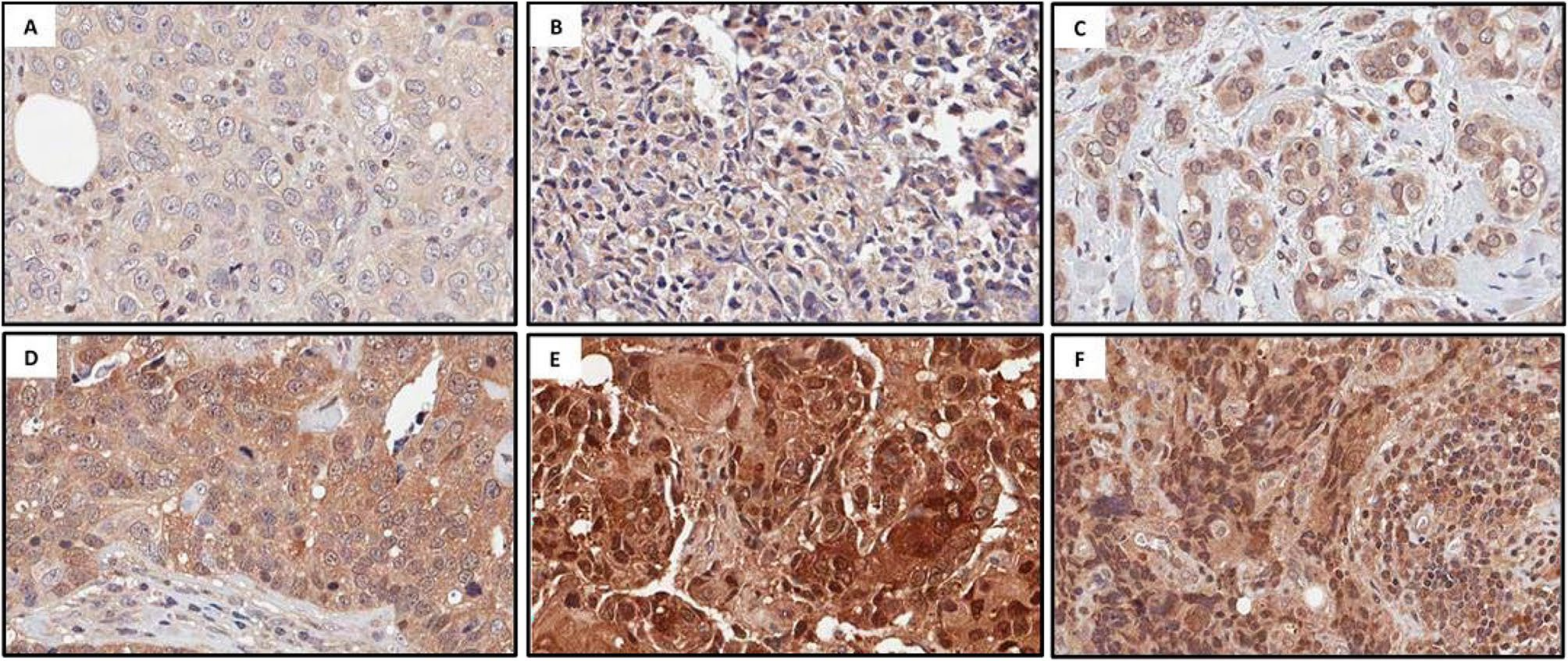
Representative images of Maspin IHC staining in human breast cancer tissue samples. The image panel shows different subcellular locations of Maspin as well as different immunostaining intensities. (**A**) and (**D**) cytoplasmic immunostaining, (**B**) and (**E**) nuclear immunostaining, C and F stromal immunostaining. (**A**), (**B**) and (**C**) low immunoreactivity and (**D**), (**E**) and (**F**) high immunoreactivity.

**Figure 2:**
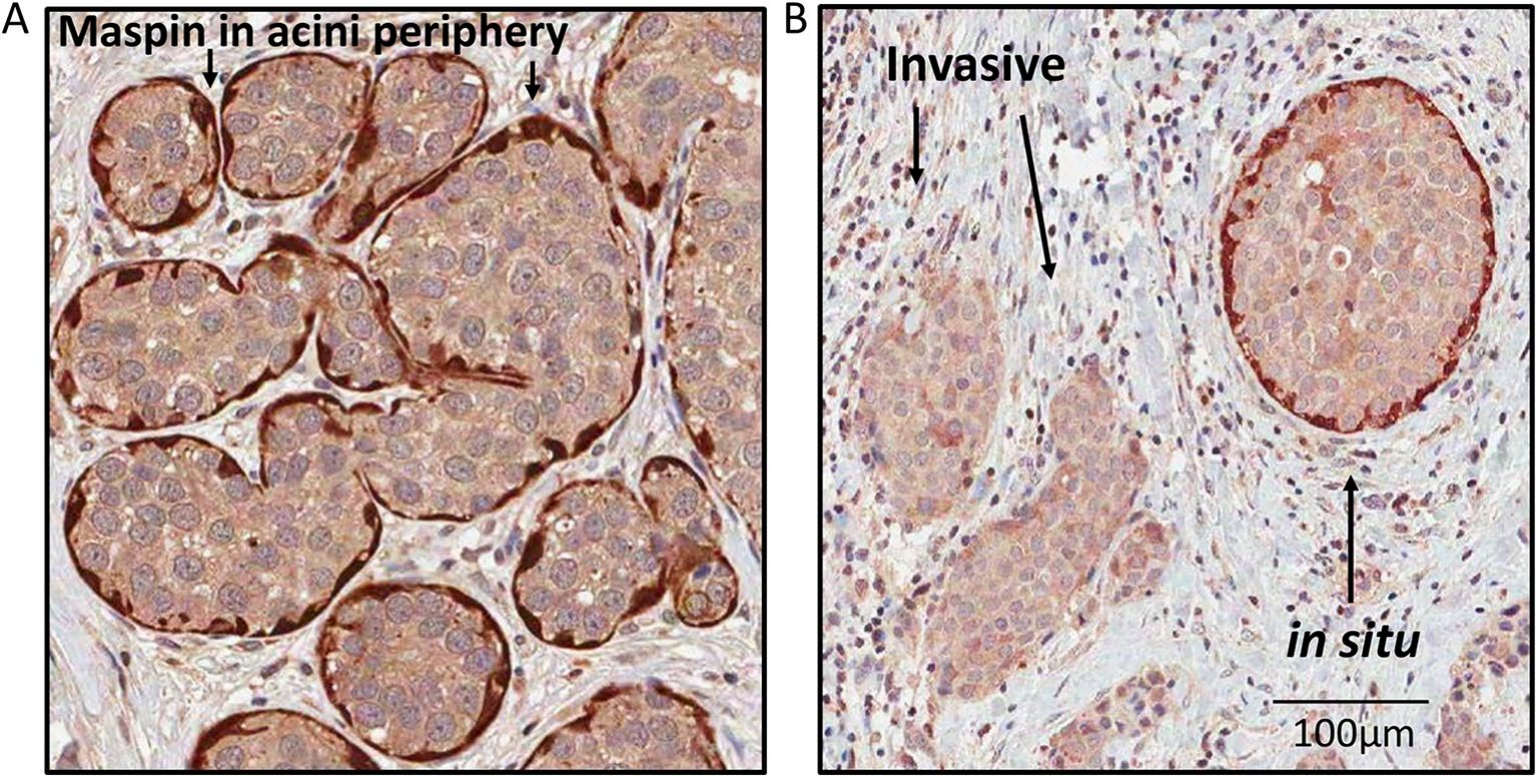
Maspin in myoepithelial cells layer. (**A**) Photomicrographs showing hyperplasic acini in breast tissue surrounded by myoepithelial cells, which express more Maspin than luminal cells. (**B**) Typical in situ component (indicated by an arrow) outlined by Maspin αV staining, whereas invasive component shows almost complete absence of peripheral Maspin. Scale bar = 100 µm. See supplementary Fig. 1 for an additional.

We evaluated the expression levels and subcellular location of the Maspin protein in our panel of primary human breast cancer tumors. The immunoreactivity of Maspin was identified predominantly in the cytoplasm and nucleus of breast cancer cells (Fig. 1). In addition, these target proteins were not restricted to tumor cells only since they were also observed in surrounding stroma and in the nuclei of inflammatory stromal cells (Fig. 1). In our visual inspection, we also noticed high Maspin expression in normal acini and hyperplasic with a preserved myoepithelial cell layer in the contour of the acini (Fig. 2A and Supp. Figure 1). In some cases, we observed Ductal Carcinoma in Situ (DCIS) components present in lesions diagnosed as Invasive Ductal Carcinoma (IDC). DCIS components were positively stained for Maspin at the periphery of the tumor cell clusters, which is consistent with the fact that the myoepithelial cells layer is preserved in these structures (Fig. 2B). In contrast, this staining pattern is lost in invasive components (Fig. 2B).

**Figure 3:**
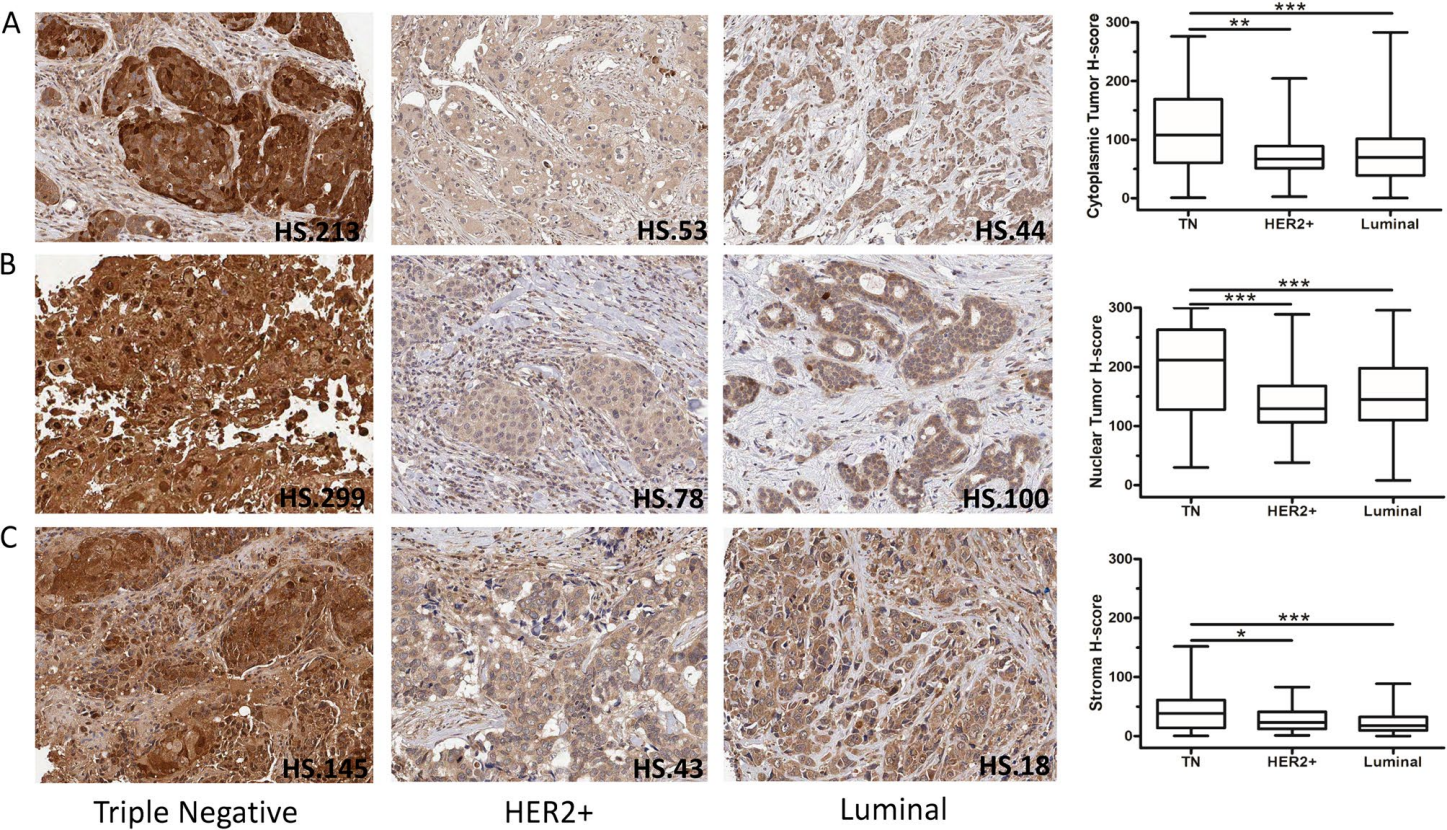
IHC staining of Maspin in samples of primary breast cancer tumors and its expression profile in different molecular subgroups. Image above representative photomicrographs of tumor tissues classified as high and low for Maspin in the TN (**A** and **D**, respectively), HER2+ (**B** and **E**, respectively) and Luminal (C and F, respectively) subtypes. Image below IHC expression levels of Maspin between the different molecular groups and according to the cytoplasmic and nuclear location in breast cancer cells (**A** and **B**, respectively). IHC expression levels of Maspin between the different molecular groups and according to the protein present in the tumor microenvironment (**C**). Statistical analyzes of the box plots were performed using the t-student or Mann–Whitney test as appropriate, with data expressed as mean ± s.e.m. (standard error). The following H-score medians were obtained for the Maspin: Cytoplasm 71.07, Nucleus 145.84, and Stroma 19.32, and subsequently, Kaplan Meyer (KM) curves were elaborated according to follow up patients’ data. *p < 0.05; **p < 0.01; ***p < 0.001.

Next, we investigated the Maspin expression levels according to the molecular subtype. In the studied cohort, Luminal represents 67.9% of cases (n = 235), HER2+, 8.7% (n = 30), and Triple Negative (Basal Like), 17.1% (n = 59). Non characterized cases are 6.4% (n = 22) as described in Supplementary Table 1. Expression data were obtained using the QuPath program and we discriminated expression according to the cytoplasmic, nuclear compartment or considering the adjacent stroma. According to the molecular subtype, Maspin protein levels were higher in both the nucleus and cytoplasm of breast cancer cells belonging to the TN tumor group compared to the other subgroups. (Cytoplasmic marking: TN vs HER2+ p = 0.0012; TN vs Luminal p < 0.0001/Nuclear Marking: TN vs HER2+ p = 0.0006; TN vs Luminal p < 0.0001) (Fig. 3). Additionally, we also detected higher protein levels of Maspin in surrounded stroma in TN tumors, in relation to the other molecular subtypes (TN vs HER2+ p = 0.0344; TN vs Luminal p < 0.0001). (TN vs HER2+ p = 0.0344; TN vs Luminal p < 0.0001) (Fig. 3). Despite comparing distinct molecular approaches.

### Association of maspin protein levels with clinical and pathological characteristics

To investigate Maspin expression in tumor cancer progression, we assessed the correlation between clinical and anatomopathological characteristics of cohort. To access Maspin expression levels in human breast cancer, we performed IHC using a tissue microarray (TMA). Expression data obtained using the QuPath program, was categorized according to subcellular location: cytoplasmic, nuclear, or secreted into the surrounding stroma. We determined a cutoff using the mean of H-score values to group samples in low (H-score under the mean) or high (H-score above mean) Maspin levels, and as described in Material and Methods. The median age of patients at the time of diagnosis was 55 years (ranging from 26 to 96 years). The tumor size in most patients was a maximum of 5 cm (72%), and the histological classification according to the Scarff-Bloom-Richardson (SBR) grading system was 13.8% grade 1, 56.9% grade 2, and 26% grade 3. In the studied cohort, 33.2% showed metastasis and the frequency of cases by molecular subtype of our cohort is comparable to a large study of distribution by molecular entities of the Brazilian female population with breast cancer (Supp. Table 1)^39^.

High Maspin protein levels were linked to increased severity in the SBR classification, specifically showing significance in Cytoplasmic marking (p = 0.047) and Stromal marking (p = 0.008). Additionally, greater lymph node involvement was associated with Stromal marking (p = 0.05) (Table 1). In addition, there were statistically significant associations between the differential expression of Maspin with the status of ER (Cytoplasmic marking: p = 0.028; Stromal marking: p < 0.0001) and PR (Nuclear marking: p = 0.025; Stromal marking: p = 0.001) and regarding the molecular subtype (Cytoplasmic marking: p = 0.037; Nuclear marking: p = 0.017; Stromal marking: p = 0.008) (Table 1).

**Table 1:** Association of the immunohistochemical expression of Maspin according to clinical-pathological data.

Variables	Category	Maspin cytoplasmic expression		p value	Maspin nuclear expression		p value	Maspin stromal expression		p value
		Low	High		Low	High		Low	High	
Age, n (%)	< 40	15 (8.7)	20 (11.6)	0.34	13 (7.5)	22 (12.7)	0.218	16 (9.2)	19 (11)	0.864
	41–59	92 (53.2)	79 (45.7)		91 (52.6)	80 (46.2)		86 (49.7)	85 (49.1)	
	≥ 60	66 (38.2)	74 (42.7)		69 (39.9)	71 (40.1)		71 (41.1)	69 (39.9)	
Hormonal Status, n (%)	Premenopause	111 (64.2)	107 (61.8)	0.656	109 (63)	109 (63)	0.098	109 (63)	109 (63)	**0.0001**
	Postmenopause	62 (35.8)	66 (38.2)		64 (37)	64 (37)		64 (37)	64 (37)	
Tumor Size, n (%)	≤ 2 cm	31 (18.1)	30 (17.5)	0.883	34 (20)	27 (15.7)	0.375	36 (20.9)	25 (14.7)	0.256
	2.1 cm–5 cm	99 (57.9)	96 (56.1)		98 (57.6)	97 (56.4)		97 (56.4)	98 (57.7)	
	> 5 cm	41 (24)	45 (26.4)		38 (22.4)	48 (27.9)		39 (22.7)	47 (27.6)	
Lymph node Involvement, n (%)	pN0	61 (35.3)	60 (35.1)	0.717	57 (32.9)	64 (37.4)	0.112	68 (39.3)	53 (31)	0.05*
	pN1	56 (32.4)	57 (33.3)		64 (37)	49 (28.7)		60 (34.7)	53 (31)	
	pN2	40 (23.1)	33 (19.3)		39 (22.6)	34 (19.9)		33 (19.1)	40 (23.4)	
	pN3	16 (9.2)	21 (12.3)		13 (7.5)	24 (14)		12 (6.9)	25 (14.6)	
Presence of Metastasis, n (%)	pM0	165 (95.4)	163 (94.8)	0.794	164 (95.3)	164 (94.8)	0.813	166 (96)	162 (94.2)	0.448
	pM+	8 (4.6)	9 (5.2)		8 (4.7)	9 (5.2)		7 (4)	10 (5.8)	
Scarff-Bloom-Richardson, n (%)	Grade 1	29 (17.4)	17 (10.3)	0.047*	30 (17.9)	16 (9.7)	0.097	27 (16.1)	19 (11.5)	0.008**
	Grade 2	101 (60.4)	96 (57.8)		95 (56.5)	102 (61.8)		108 (64.3)	89 (53.9)	
	Grade 3	37 (22.2)	53 (31.9)		43 (25.6)	47 (28.5)		33 (19.6)	57 (34.5)	
Nuclear Grade, n (%)	Grade 1	2 (1.2)	1 (0.6)	0.412	2 (1.2)	1 (0.6)	0.195	2 (1.2)	1 (0.6)	0.319
	Grade 2	60 (36.6)	49 (30.1)		62 (37.6)	47 (29)		62 (37.1)	47 (29.4)	
	Grade 3	102 (62.2)	113 (69.3)		101 (61.2)	114 (70.4)		103 (61.7)	112 (70)	
Clinical Internship, n (%)	Stage I	9 (5.20	10 (5.8)	0.983	7 (4)	12 (6.9)	0.356	9 (5.2)	10 (5.8)	0.09
	Stage II	93 (53.8)	90 (52)		99 (57.2)	84 (48.6)		103 (59.5)	80 (46.2)	
	Stage III	63 (36.4)	64 (37)		59 (34.1)	68 (39.3)		54 (31.3)	73 (42.2)	
	Stage IV	8 (4.6)	9 (5.2)		8 (4.7)	9 (5.2)		7 (4)	10 (5.8)	
Estrogen Receptor, n (%)	Negative	43 (25.7)	61 (37)	0.028*	45 (27.1)	59 (35.5)	0.098	37 (22.3)	67 (40.4)	< 0.0001****
	Positive	124 (74.3)	104 (63)		121 (72.9)	107 (64.5)		129 (77.7)	99 (59.6)	
Progesterone Receptor, n (%)	Negative	76 (45.2)	91 (54.5)	0.09	73 (43.7)	94 (56)	0.025*	68 (40.7)	99 (58.9)	< 0.001***
	Positive	92 (54.8)	76 (45.5)		94 (56.3)	74 (44)		99 (59.3)	69 (41.1)	
HER2, n (%)	Negative	121 (85.2)	126 (84.6)	0.878	114 (82)	133 (87.5)	0.192	125 (88.7)	122 (81.3)	0.082
	Positive	21 (14.8)	23 (15.4)		25 (18)	19 (12.5)		16 (11.3)	28 (18.7)	
Molecular Subtypes	Triple Negative	20 (14.3)	39 (26.5)		19 (13.9)	40 (26.7)	0.017*	20 (14.4)	39 (26.4)	
	HER2+	16 (11.4)	14 (9.5)	0.037*	18 (13.1)	12 (8)		11 (7.9)	19 (12.8)	0.008**
	Luminal	104 (74.3)	94 (64)		100 (73)	98 (65.3)		108 (77.7)	90 (60.8)	

HER2, human epidermal growth factor receptor 2.

p-values obtained by Pearson’s chi-square test or Fisher's exact test. *p < 0.05; **p < 0.01; ***p < 0.001; ****p < 0.0001. Fisher was applied when 25% or more of the expected value was less than 5.

Significant values are in bold.

### Prognostic value of maspin protein level on breast cancer patients' survival

**Figure 4:**
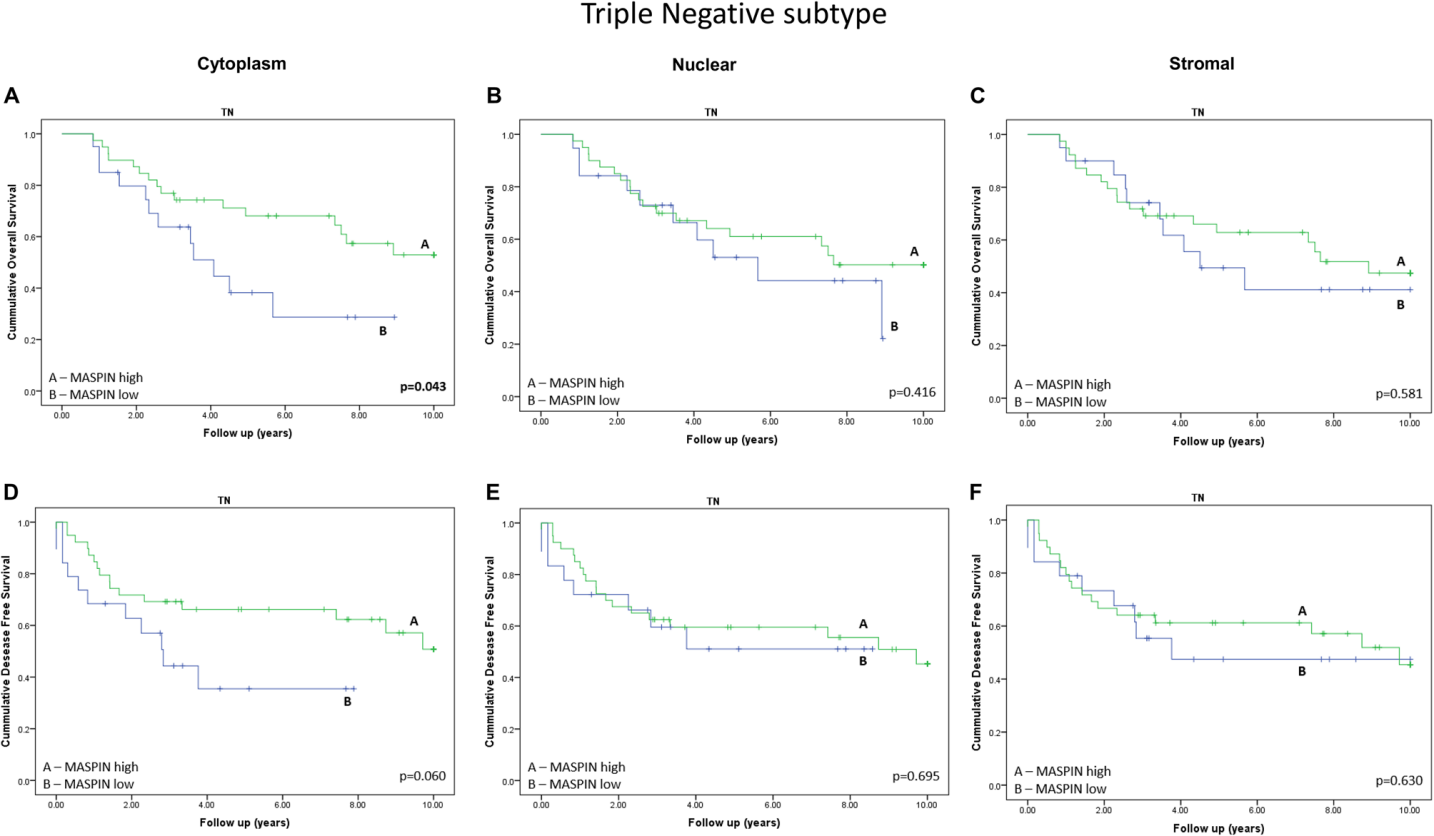
Kaplan–Meier curves for overall survival (OS) and disease-free survival (DFS) of patients with breast cancer of the TN subtype stratified according to the Maspin expression. The OS curves (**A**, **B** and **C**) and DFS curves (**D**, **E** and **F**) were calculated for each group of patients. All cases were classified as high or low expression for Maspin. (**A**) and (**D**) Evaluation of cytoplasmic immunostaining. (**B**) and (**E**) Evaluation of nuclear immunostaining. (**C**) and (**F**) Stromal immunostaining assessment.

Next, we assessed the prognostic value of Maspin protein level in relation of breast cancer patient’s outcome data in Kaplan Meyer curves analysis. Initially, we did not detect any significant differences in the protein levels of Maspin affecting the survival of breast cancer patients when analyzing all molecular subtypes simultaneously (Supp. Figure 2). Likewise, we also did not find significant differences in Maspin expression in the Luminal and HER2 + subtypes. (Supp. Figure 3 and Supp. Figure 4). However, in TN subtype tumors, we observed that patients with lower Maspin expression had a worse prognosis for cancer specific survival (Cytoplasmic marking: OS p = 0.043; DFS p = 0.06) (Fig. 4). We found no significant correlation with the expression of nuclear Maspin or surrounding stroma (Fig. 4).

### Maspin mRNA levels and breast cancer patients' survival

**Figure 5:**
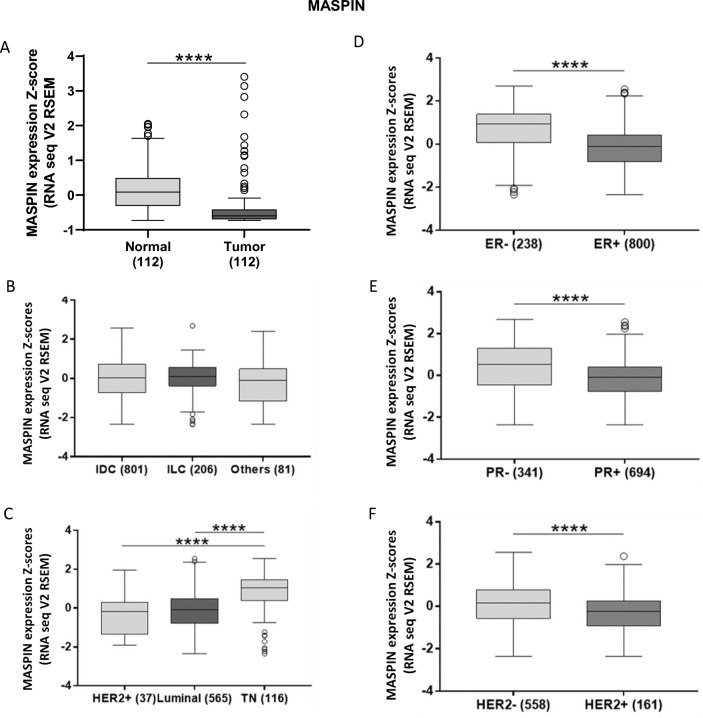
Expression profile of Maspin in different tissue and molecular contexts. (**A**) Differential expression of Maspin in normal and tumoral breast tissue, (**B**) in different histopathological subtypes and (**C**) in different molecular subtypes of breast cancer. On the right, expression profile of Maspin in breast cancer according to classical breast cancer biomarkers. (**D**) Expression of Maspin and the estrogen receptor. (**E**) Maspin and the progesterone receptor, and (**F**) expression of Maspin and HER2, respectively. *p < 0.05; **p < 0.01; ***p < 0.001; ****p < 0.0001. Results based on TCGA data.

In silico analyses were conducted using TCGA RNA-seq data to evaluate the expression of *SERPINB5* gene, official name of the gene that express Maspin protein, in tumor samples and normal tissue. It was observed that this gene is differentially expressed in tumor tissue compared to normal breast samples, while Maspin mRNA levels were significantly reduced in tumors (p < 0.0001) (Fig. 5A). Corroborating the in silico data, our Western Blot evaluation revealed that Maspin mRNA *levels* was significantly lower in breast tumor cell lines when compared to normal cell lines (Supp. Figure 4). No significant differences were observed between the different histological subtypes and the mRNA levels of Maspin (Fig. 5B). The stratification of tumors in the different subtypes demonstrated that Maspin mRNA levels has a significant association with breast cancer subtypes and that TN tumors is the group with the highest expression levels (p < 0.0001) (Fig. 5C).

Then, we also classified the patients according to different clinicopathological parameters and based on estrogen, progesterone and HER2 receptors status. Maspin mRNA levels were significantly reduced in patients with ER (p < 0.0001), PR (p < 0.0001) and HER2 (p < 0.0001) positive tumors (Fig. 5D, E and F) and there was a significant association between Maspin mRNA levels and age (p < 0.0001), hormonal status (p < 0.0001), TP53 status (p = 0.002) and TN status (p < 0.0001) (Supplementary Table 2).

**Figure 6:**
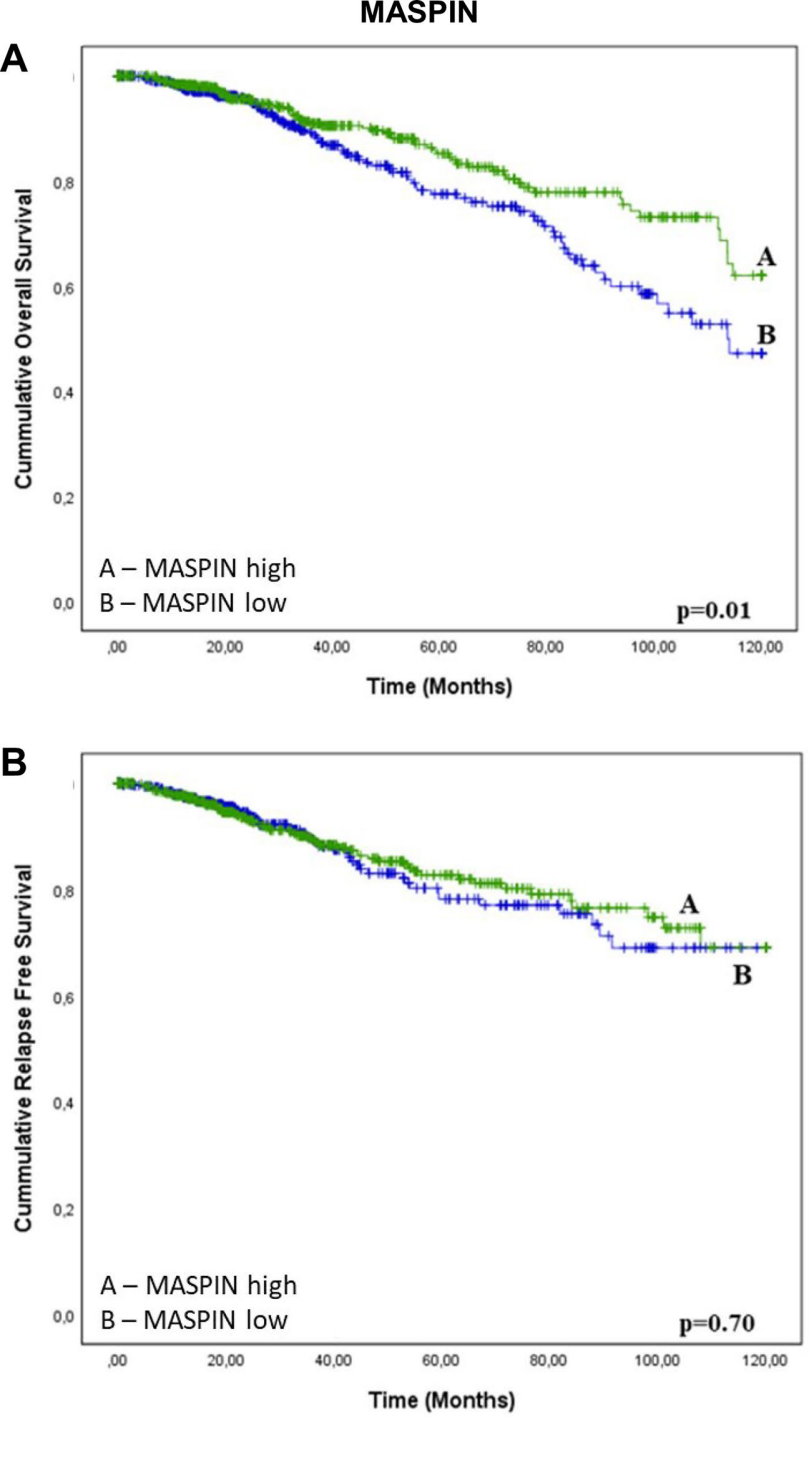
Kaplan–Meier curves for breast cancer patient survival as a function of Maspin mRNA expression. Kaplan–Meier curves for overall survival (OS) and relapse-free survival (RFS) of breast cancer patients stratified according to mRNA of Maspin. All patients are included in this analysis. The analyses are conducted using the on TCGA Firehose Legacy breast cancer patients. ****p < 0.0001. Results based on TCGA data.

We assessed the prognostic significance of Maspin mRNA level in 1088 patients from TCGA database. The results show that low mRNA levels of Maspin are associated to a reduced overall survival conferring a worse prognosis (Fig. 6A). There was no association between Maspin mRNA levels and relapse free survival (Fig. 6B).

## Discussion

Cancer biomarkers are valuable clinical tools that can be used for diagnostic and prognostic purposes, as well as predictive therapeutic approaches. Despite advances in cancer biology research, coupled with the use of "omic" technologies, only a few candidates have been successfully adopted in the routine clinic^40^. For breast cancer, this problem is even greater due to the surprising inter- and intratumoral genetic heterogeneity^41,42^.

Maspin is a member of the Serpin family, which has pleiotropic action and a great diversity of molecular targets^43,44^. Despite initially being found in breast tissue^45^, hence its name Mammary serine protease inhibitor, it is widely expressed in different types of homeostatic epithelial cells^44,46^. Among the various cellular processes influenced by Maspin, adhesion, migration, invasion, proliferation, cell death and oxidative stress deserve special attention, because are intricate to neoplastic progression^43,44,47^. In breast tissues, Maspin is highly expressed in normal epithelial cells, especially in myoepithelial cells^47–49^. However, Maspin expression levels in neoplasms show contradictory findings. If on the one hand, there is data in the literature demonstrating that Maspin expression is down-regulated in breast^45^, prostate^50^, gastric^15^ and melanoma^51^ cancers^46–49^, other evidence demonstrated over-expressed in pancreatic^52^, gallbladder, colorectal, and thyroid cancers^50–53^. The controversy may even be greater considering that some studies show that Maspin expression may be biphasic, being silenced at early stages of tumorigenesis and re-expressed in the metastatic phase^47^, suggests that Maspin has versatile biological functions under different pathophysiological context, which does not make its suggestion as a biomarker pragmatic. In this work, our findings showed no correlation between Maspin protein level and survival of breast cancer patients in the studied cohort, when analyzed collectively. What had also been reported in the literature^53^. However, our TCGA analyses demonstrated that Maspin mRNA levels in neoplastic breast tissues are lower when compared to their non-tumor counterparts. We could not perform a similar analysis at the protein level using our TMA cohort because the tissue deposited in the blocks did not comprise samples of normal breast tissue from the respective patients. Despite comparing distinct molecular approaches—using mRNA or protein data for patient stratification in survival curve analysis from two different populations—, the observed discrepancy between protein and mRNA expression levels can potentially be attributed to variations in the dynamics of RNA expression and protein translation, degradation, as well as different post-transcriptional regulatory mechanisms. Should this pattern persist within the same population when comparing mRNA/protein parameters, it highlights the independence of these processes and their susceptibility to distinct regulatory influences^54,55^. Furthermore, it must be considered that these analyses were carried out on different cohorts, which may reflect heterogeneous genetic backgrounds.

Intriguingly, evidence in the literature indicating that, more than the absolute expression level of Maspin, its subcellular location correlates better with tumor suppression and prognosis^22,56,57^. Maspin protein has already been found in the cytoplasmic and nuclear compartment^23,58^, as well as a cell surface-associated protein^20^. Furthermore, it has also been documented to be secreted extracellularly^46,59^. Surprisingly, subcellular location seems to have different prognostic values, that in the nucleus it may be associated with good prognosis^60–62^, whereas cytoplasmic localization correlates with poor prognosis^63–65^. This discrepancy can be explained by Maspin’s interaction with different molecular targets, acting either as a suppressor or promoter of the neoplastic process depending on the cellular circumstances^43,66,67^. In our work, by visually inspecting the images relating to the cases in our TMA cohort, Maspin was detected by IHC in the nucleus, cytoplasm, as well as secreted in the tumor microenvironment of these tissues, corroborating with previous published data^23,46,59^. Interestingly, in cases that presented mammary gland components (or DCIS component associated) with preserved epithelial architecture, Maspin protein level appears to be juxtaposed with the myoepithelial cell layer, corroborating with previous publications^49–51^. Despite this, our results did not demonstrate statistically significant correlations between nuclear, cytoplasmatic or stromal Maspin protein level and the survival rates or incidence of metastases in the cohort patients analyzed collectively. So, we proceeded to analyze according to the molecular subtype.

Classification of breast carcinomas into subtypes according to the expression of molecular markers, mainly ER, PR and HER-2, brought highly significant correlations with clinical outcomes, including overall survival and recurrence free survival^68,69^. For decades HER-2 has been used as a breast cancer biomarker associated with alteration in gene expression, and by extension, predictor of Trastuzumab efficacy^70^. However, only a limited proportion of breast cancer patients are HER-2+, and even in these, there is a chance that the expression will lose consistency and they will become refractory to treatment^71,72^. Likewise, hormone therapy is conditioned on the detection of ER and PR receptors in luminal subtypes^73^. For the triple-negative breast cancer subtype, which is known to have worse outcome compared to other breast cancer subtypes^74^, remains without specific treatment guidelines^75^ and identifying markers as well^69^. Our clinicopathological evaluation demonstrated that positive tumor expression of both ER and PR was significantly associated with lower Maspin expression, and it is precisely these markers that define the molecular subtypes. This corroborates previous data in the literature, since ER has been described to suppress Maspin expression^64,65^. Furthermore, our results shows that Maspin is more expressed in the TN subtype when compared to Luminal and HER2+ subtypes. Corroborating this finding, our silico analyses using TCGA RNA-seq data show higher levels of Maspin mRNA in TN subtypes when compared to other subtypes. If Maspin protein and transcript levels are reduced in the TN subtype, we next asked whether it could have any prognostic value in this subtype. Our Kaplan–Meier curves indicate that TN patients with lower cytoplasmatic Maspin had a worse prognosis for Overall survival. Even so, we recognize that the value of this observation for the prognosis is limited.

There has been debate about the involvement of the host stroma and a diversity of non-tumor cell populations in tumor progression^76^. In this context, several mechanisms can contribute synergistically, such as molecules that change the pattern of gene expression in the tumor^77^ or being improperly secreted^78,79^, as well as heterotypic cell interactions^80^ mediated by mediators such as growth factors^81^, cytokines^82^, exosomes^83^ and others^84^, that contributes for maintenance of immunosuppressive microenvironment, pro-tumor, with high potential to be refractory to conventional therapies^85^. When analyzing the images of the cases in our cohort, we noticed the immunostaining of both Maspin in regions corresponding to the stroma. When analyzing h-score of Maspin staining in the stroma, our results indicated that is greater in the TN, when compared to the Luminal and HER2 subtypes. Evidence in the literature indicates that Maspin is predominantly cytoplasmic^86^, with some membrane or nucleus association^87^, but which may also be partially secreted^46,70^. Nevertheless, Maspin at the epithelium-stroma interface, which most likely corresponds to myoepithelial cells, is an indicator of a better clinical outcome^88^. Several evidence in the literature have shown that the subcellular location of Maspin may have greater prognostic value in some types of cancer than its absolute expression measure^21,56,67,89,90^. On the other hand, it is yet unclear the consequences of the presence of Maspin in the extracellular matrix adjacent to tumors. Although our data do not explain the discrepancy between the highest Maspin expression in TN patients versus the worst prognosis of those with low expression in this subtype, changes in subcellular location or even its secretion could be speculated. Even so, our data suggested that stromal Maspin was related to greater lymph node involvement. Further investigations will be necessary to better elucidate its involvement in tumor progression, as well as its possible use as a prognostic indicator.

## Closing remarks

Our TMA expression data did not detect statistically significant relationships between Maspin expression levels and indices of overall survival when to the entire cohort analyzed collectively. However, when divided by molecular subtype, Triple negative (TN) presents alterations in the expression levels of Maspin, in both of its mRNAs, and at the protein level. Although TN breast cancer has Maspin levels increased compared to other Luminal and HER2 subtypes, our results shows that downregulation in TN had a poor prognosis. Collectively, the Maspin expression data are not consistent for indicating a prognostic marker in breast cancer, but they demonstrate that there is a change in the expression profile in the TN subtype.

### Supplementary Information

Supplementary Table 1.

Supplementary Table 2.

Supplementary Figures.

## Data Availability

Please contact corresponding author (Otto Cerqueira: ottoluix@alumni.usp.br) for data requests.
