# Supplementary material for: Prognostic value of Maspin protein level in patients with triple negative breast cancer
Source: Sci Rep. 2024 Jul 10;14:15982. doi: 10.1038/s41598-024-53870-y (PMC11237076; doi:10.1038/s41598-024-53870-y)
Supplement: Supplementary file 3 — Supplementary Figures. [file 41598_2024_53870_MOESM3_ESM.pptx]

## Slide 1
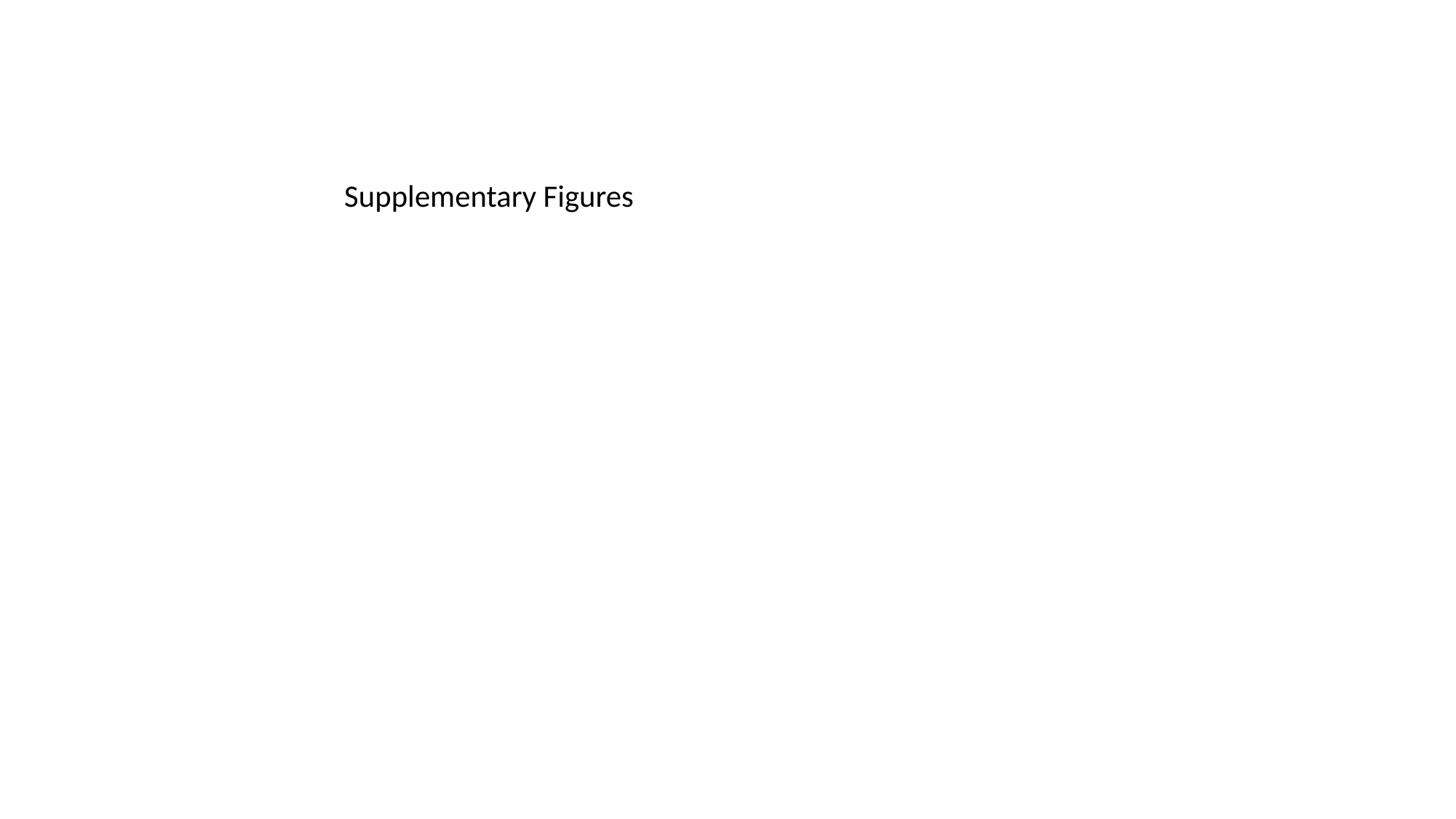

Supplementary Figures

## Slide 2
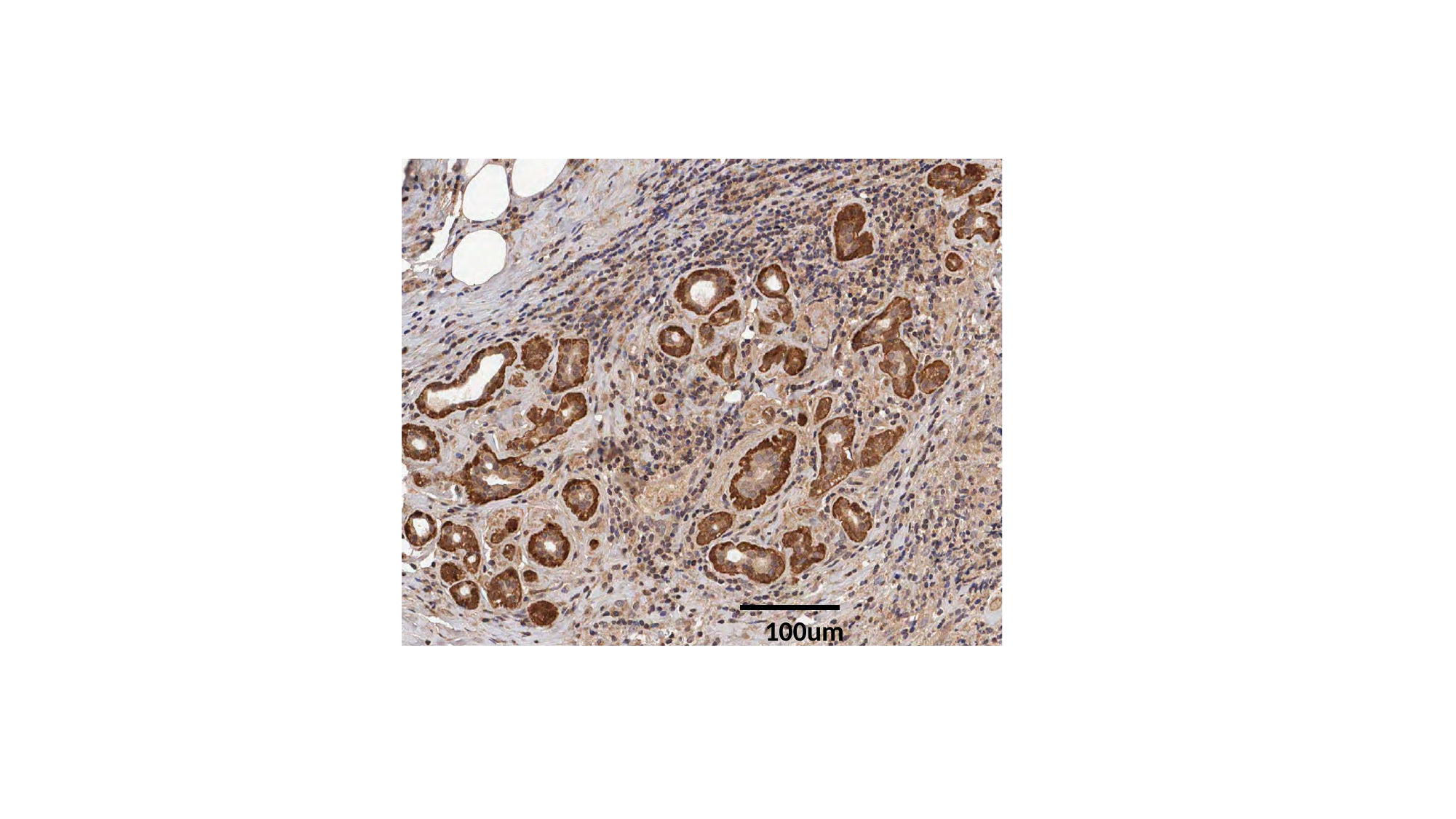

100um

## Slide 3
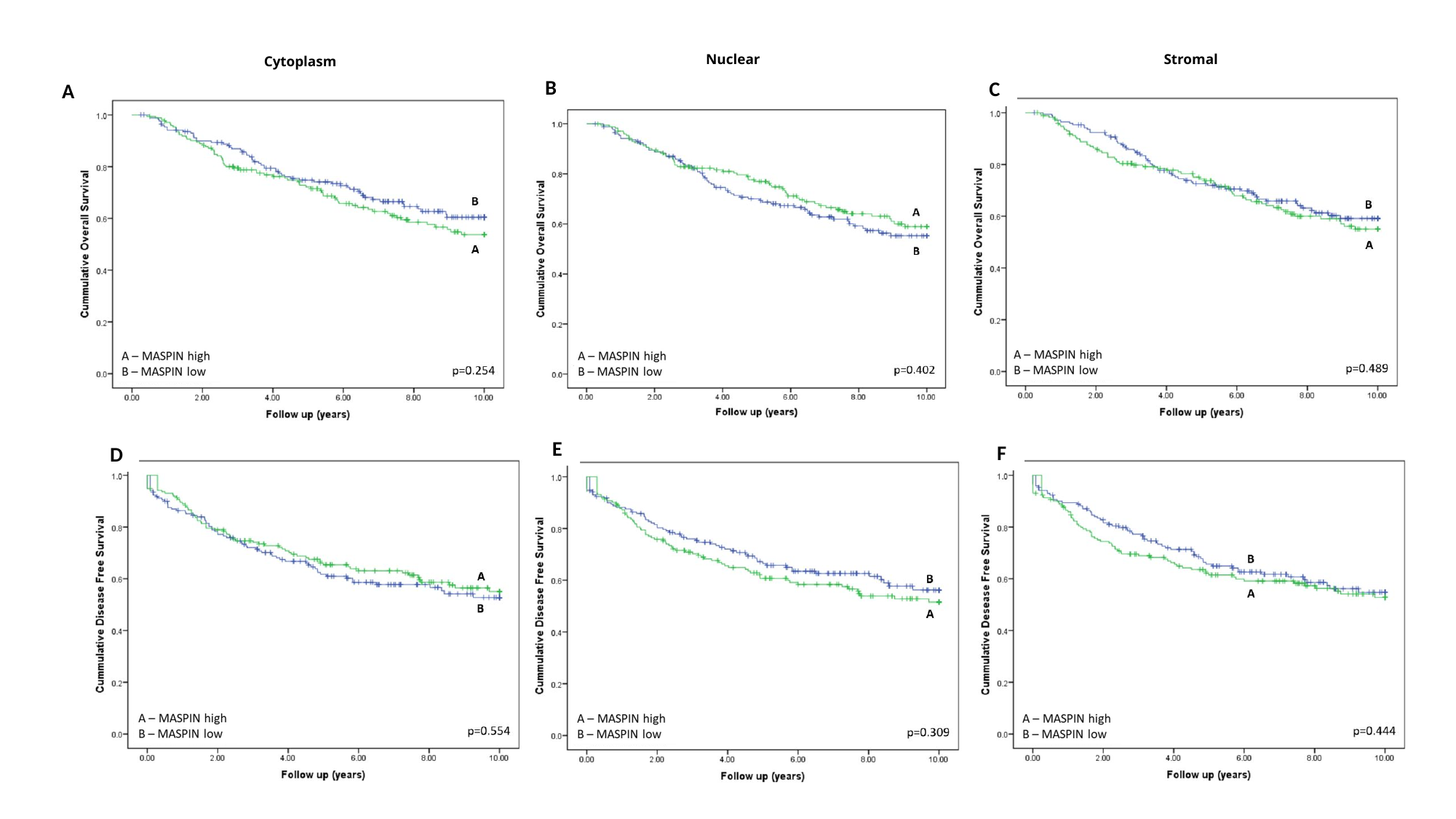

Nuclear
Stromal
Cytoplasm
B
C
E
F
D
A

## Slide 4
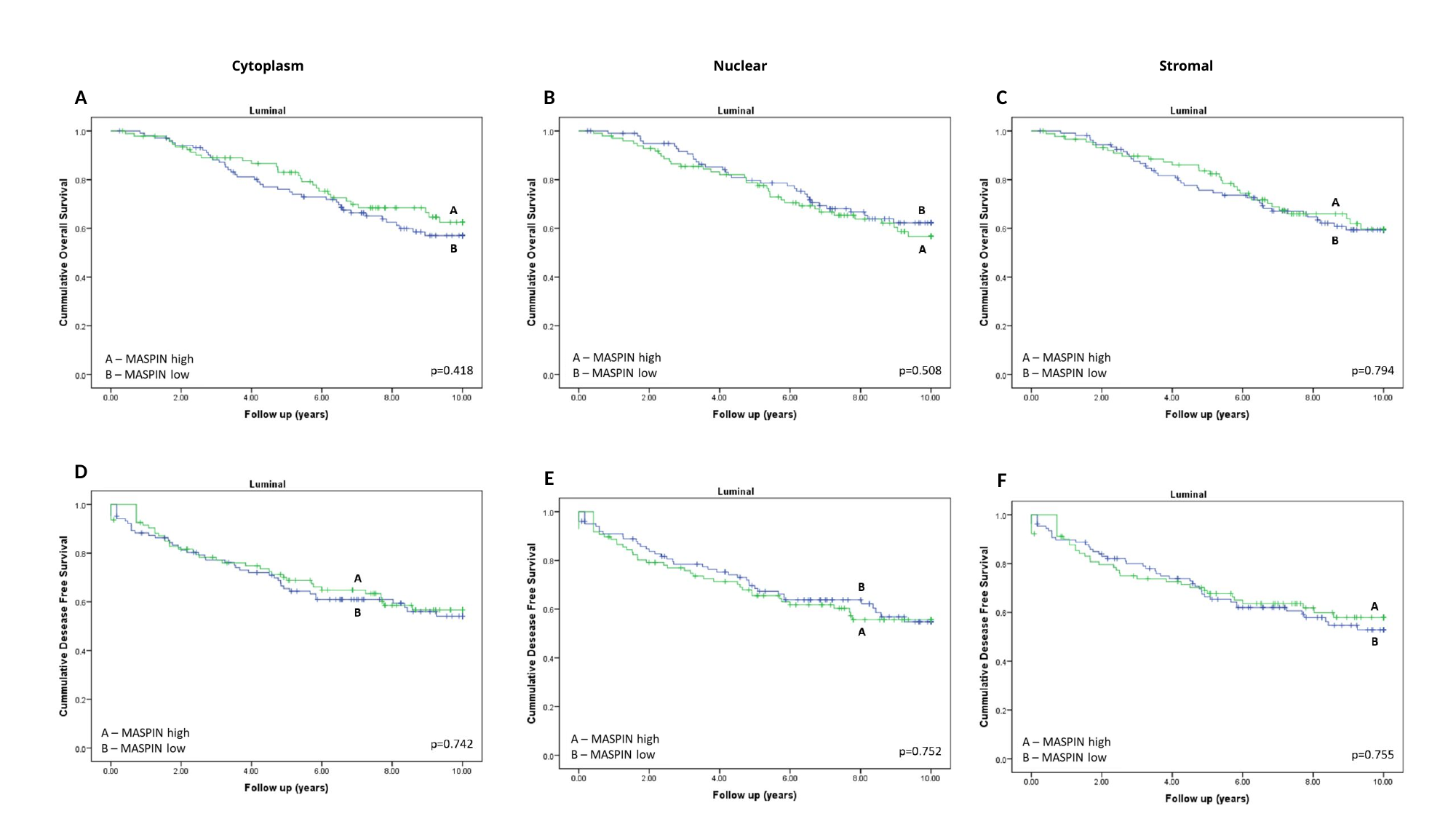

Cytoplasm
Nuclear
Stromal
C
D
E
F
A
B

## Slide 5
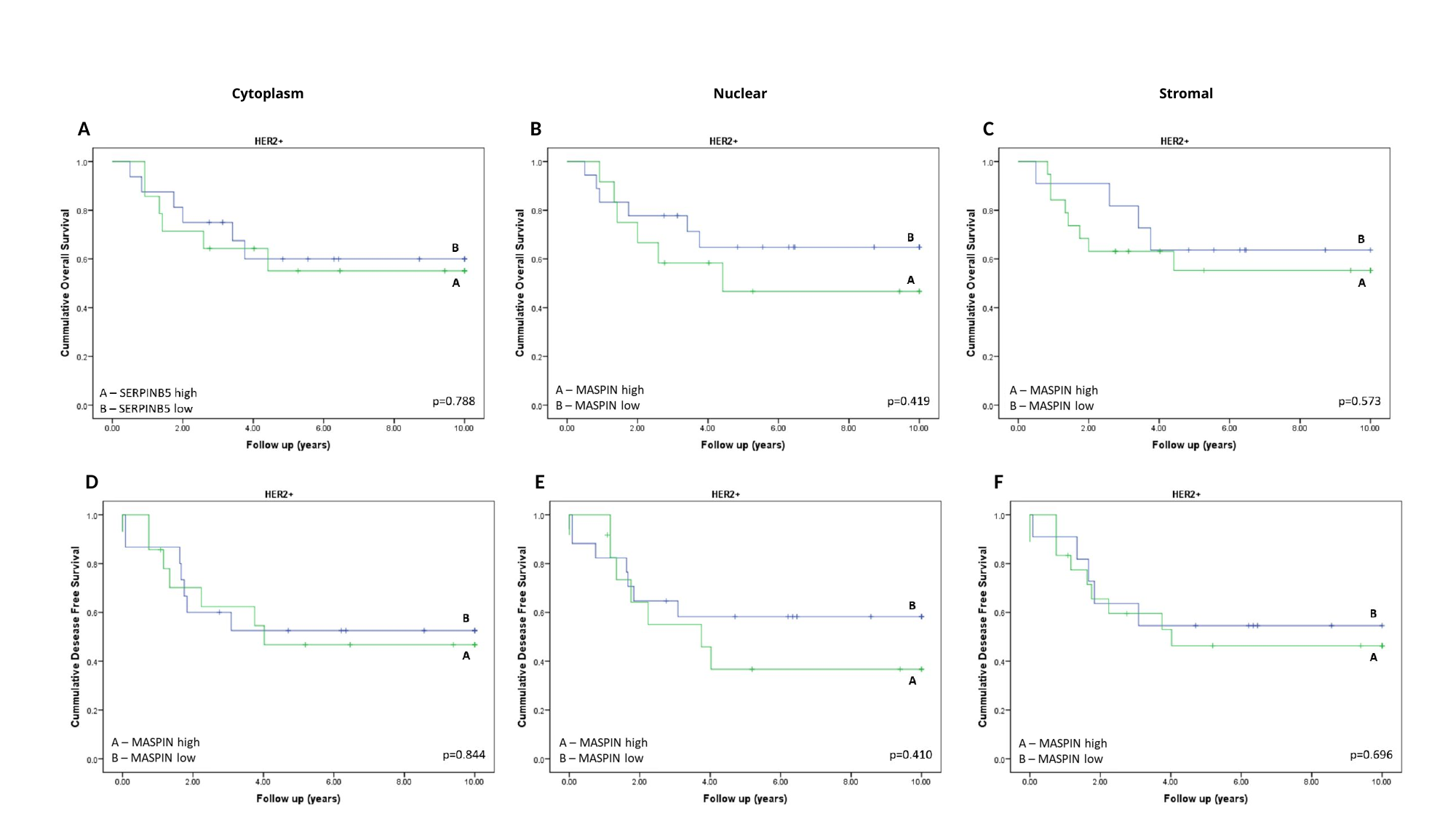

Cytoplasm
Nuclear
Stromal
C
D
E
F
A
B

## Slide 6
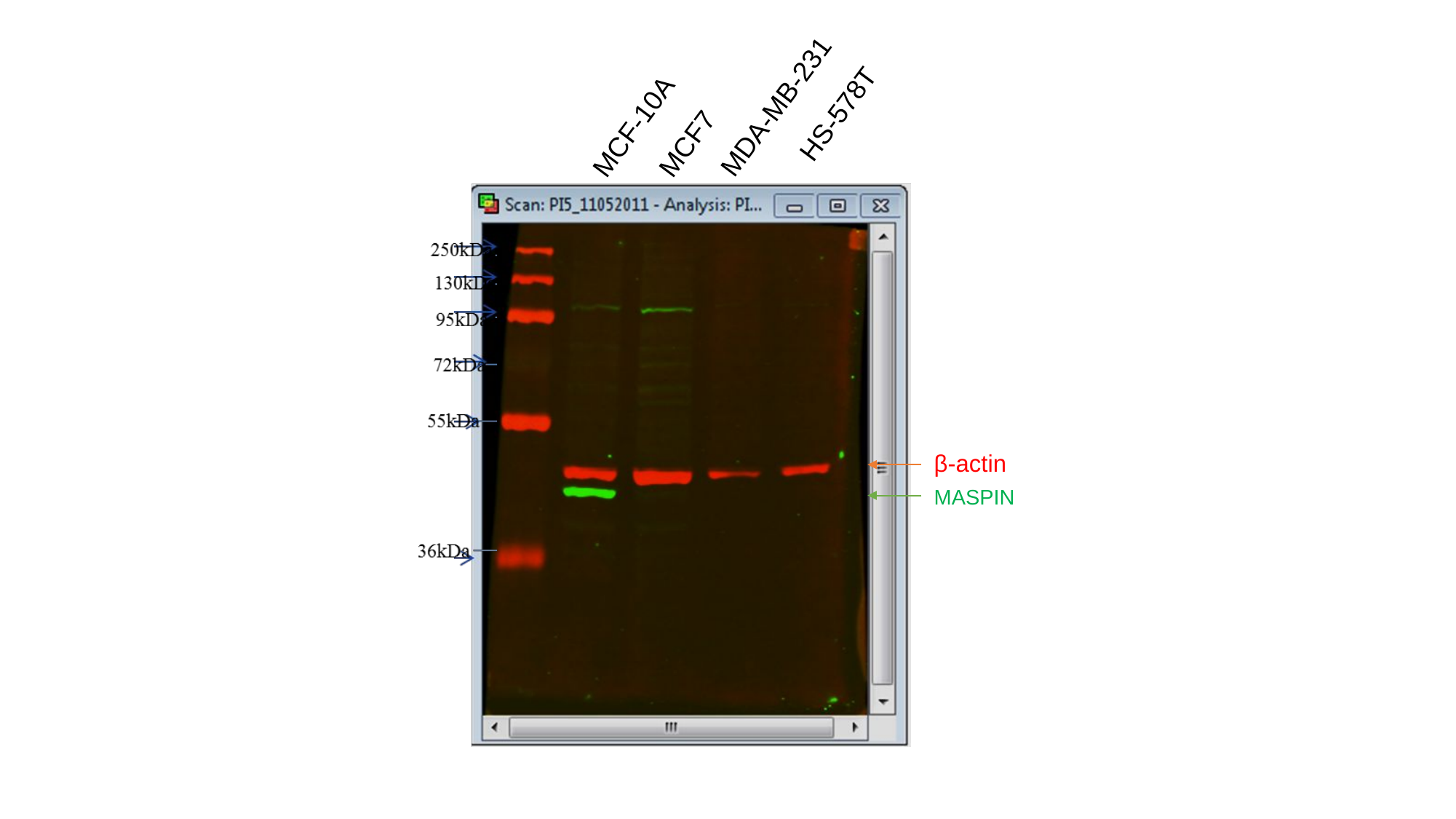

MDA-MB-231
HS-578T
MCF-10A
MCF7
β-actin
MASPIN
